# Morphology and immunolocalization of aquaporins 1 and 9 in the agouti (*Dasyprocta azarae*) testis excurrent ducts

**DOI:** 10.1590/1984-3143-AR2021-0070

**Published:** 2021-11-04

**Authors:** Bruno Cesar Schimming, Leandro Luis Martins, Fabrício Singaretti de Oliveira, Patrícia Fernanda Felipe Pinheiro, Raquel Fantin Domeniconi

**Affiliations:** 1 Departamento de Anatomia, Universidade Estadual Paulista, Botucatu, São Paulo, Brasil; 2 Departamento de Anatomia, Universidade Estadual de Londrina, Londrina, PR, Brasil; 3 Departamento de Morfologia e Fisiologia Animal, Universidade Estadual Paulista, Jaboticabal, São Paulo, Brasil

**Keywords:** aquaporins, morphology, rodents, epididymis, efferent ducts

## Abstract

This study investigated the morphology and immunoexpression of aquaporins (AQPs) 1 and 9 in the rete testis, efferent ducts, epididymis, and vas deferens in the Azara’s agouti (*Dasyprocta azarae*). For this purpose, ten adult sexually mature animals were used in histologic and immunohistochemical analyses. The Azara’s agouti rete testis was labyrinthine and lined with simple cubic epithelium. Ciliated and non-ciliated cells were observed in the epithelium of the efferent ducts. The epididymal cellular population was composed of principal, basal, apical, clear, narrow, and halo cells. The epithelium lining of vas deferens was composed of the principal and basal cells. AQPs 1 and 9 were not expressed in the rete testis. Positive reaction to AQP1 was observed at the luminal border of non-ciliated cells of the efferent ducts, and in the peritubular stroma and blood vessels in the epididymis, and vas deferens. AQP9 was immunolocalized in the epithelial cells in the efferent ducts, epididymis and vas deferens. The morphology of Azara’s agouti testis excurrent ducts is similar to that reported for other rodents such as *Cuniculus paca*. The immunolocalization results of the AQPs suggest that the expression of AQPs is species-specific due to differences in localization and expression when compared to studies in other mammals species. The knowledge about the expression of AQPs in Azara’s agouti testis excurrent ducts is essential to support future reproductive studies on this animal, since previous studies show that AQPs may be biomarkers of male fertility and infertility.

## Introduction

The absorptive and secretory capacities of the epithelial cells lining the male excurrent ducts create an appropriate microenvironment for the sperm maturation process. Movement of fluid through the epithelium of the testis excurrent ducts is necessary for the concentration of spermatozoa, which is pivotal for fertility. Fluid regulation is an important process of promoting sperm differentiation and maturation (Pelagalli et al., 2019). Between 50 and 80% of the testicular fluid is reabsorbed in the efferent ducts (Clulow et al., 1994), but a considerable reabsorption activity also occurs in the epididymis (Da Silva et al., 2006). This reflects a significant increase in sperm concentration when the fluid transits towards the distal regions of the epididymis, establishing a hypertonic luminal fluid (Levine and Marsh, 1971).

The aquaporins (AQPs) are a family of intrinsic membrane proteins, present in many cell types involved in fluid transport (Verkman and Mitra, 2000; Agre, 2004). The transport of water and solute, which occurs in the efferent ducts and the epididymis, is necessary to establish a proper luminal environment for the maturation, concentration, and storage of spermatozoa (Belleannée et al., 2009; Belleannée et al., 2012). It is believed that AQPs provide a mechanism with low energy expenditure for the rapid movement of water through the epithelium (Hermo et al., 2008); therefore, they are essential for the regulation of cell volume and transepithelial water transport, thus contributing to body homeostasis (Verkman, 2002).

Thirteen isoforms of AQPs have been identified in mammalian cells, namely AQPs 0 to 12 (Brown, 2017; Carrageta et al., 2020). According to their permeability characteristics, the AQPs are divided into two major groups: the AQPs (AQPs 0, 1, 2, 4, 5, 6, and 8) that are highly selective for water, and the aquaglyceroporins that are permeable to water and glycerol (AQPs 3, 7 and 10) or water and large solutes (AQP9) (Da Silva et al., 2006).

There are many studies on the AQP-1 and -9 expressions in the male reproductive tract. The AQP-1 has been considered a protein responsible for water reabsorption since it has been found in non-ciliated cells of the human efferent ducts and in the epididymis (Nicòtina et al., 2005; Oberska and Michalek, 2021), and concerning AQP9, it has been suggested that this AQP could be involved in the movement of water and small solute molecules through the male reproductive tract (Da Silva et al., 2006; Oberska and Michalek, 2021).

The *Dasyprocta* sp. is a wild rodent that belongs to the order Rodentia, the family Dasyproctidae, and the genus Dasyprocta. Several species of this genus appear in Brazilian territory (Lange and Schmidt, 2014). The species *Dasyprocta azarae* (Azara’s agouti) is a medium-sized rodent with herbivorous, frugivorous, terrestrial, and diurnal habits. This animal lives in South America, including Brazil, Argentina, and Paraguay (Eisenberg and Redford, 1999). The ecological role of the Azara’s agouti can be exemplified by the fact that this animal has been considered an important seed disperser (Silva et al., 2013; Praxedes et al., 2018). Moreover, this mammal represents an alternative source of animal protein for the population, besides serving as food for other wild animals (Arroyo et al., 2014; Castelo et al., 2015). Given this zootechnical potential, raising of Azara’s agouti in captivity preserve the species and its use as a production animal.

The morphology and biology of reproduction in the male agouti have been investigated. Some studies have performed analyses of the ultrastructure of the epididymis and vas deferens at various stages of sexual development in *Dasyprocta* spp. (Arroyo et al., 2014), macroscopic anatomy of the male reproductive system (Mollineau et al., 2006), and male accessory glands (Mollineau et al., 2009). Penile erection and semen collection by electroejaculation also have been described (Mollineau et al., 2012; Martinez et al., 2013; Castelo et al., 2015). The spermatogenesis and spermatozoa of *Dasyprocta* spp. also have been described at the ultrastructural level (Arroyo et al., 2017). However, by light microscopy, there are no reports on the morphology presented in the rete testis, efferent ducts, epididymis, and vas deferens in *Dasyprocta* sp., particularly in the species *D. azarae*. Thus, the aim of this study was to evaluate the morphology, by light microscopy, and identify the presence of AQPs -1 and -9 in the testis excurrent ducts of Azara’s agouti since there is a total absence of information on the immunolocalization of AQPs in the testis excurrent ducts of this animal and in this way, contribute to new knowledge about the reproductive biology of this Neotropical rodent.

## Materials and methods

### Animals and tissues

In this study, ten Azara’s agoutis (*Dasyprocta azarae*) were used, males and adults, from the Municipal Zoo, Catanduva, in the state of São Paulo, Brazil. The testis excurrent ducts were obtained during castration surgery performed with the objective of population control. The protocol for anesthesia was adapted from Martinez et al. (2013). Fragments of the testis, epididymis, and initial (proximal) segment of the vas deferens were fixed in 4% paraformaldehyde for 24 h. After histological fixation, tissue samples were submitted to increasing alcohol passage and routinely included in Paraplast^TM^ (Sigma, St. Louis, MO, USA). Following inclusion, 5 μm thick histological sections were obtained for histologic and immunohistochemistry routines. The material for histological analysis was stained with hematoxylin and eosin (H&E), and Masson’s trichrome. These histological sections were used for identify the histological features of the Azara’s agouti rete testis, efferent ducts, epididymis, and vas deferens. The present study was approved by the Ethics Committee on the Use of Animals (CEUA) of the Universidade Estadual Paulista (UNESP), under protocol number 796/2015.

### Immunohistochemistry

For the immunolocalization of the AQPs, deparaffinized slices were subjected to antigen retrieval solution (Citrate buffer, pH 6.0) in the microwave. Endogenous peroxidase was pre-blocked by using 3% hydrogen peroxide (H_2_O_2_) in methanol at room temperature. The sections were washed in PBS and incubated in 3% BSA in PBS for 1 hour at room temperature to block non-specific staining, followed by overnight incubation in a moist chamber at 4°C with the relative primary antibodies (rabbit polyclonal directed against AQP-1 and AQP-9) (Biosciences Research Reagents, Temecula, CA, USA) at 1:100 dilution. The slides were subsequently washed in PBS, and a secondary antibody (Santa Cruz Biotechnology, CA, USA) was applied for 1 hour at room temperature, followed by three washes in PBS. The immunoreactive sites were visualized using diaminobenzidine tetrahydrochloride (DAB) solution, and sections were counterstained with Mayer’s hematoxylin. A positive control using rat kidneys was obtained during the same time. The negative control was performed by suppressing the primary antibody (data not shown). Staining intensities were evaluated based on subjective estimates of two of the authors and scored on a scale from + to +++. Slides were observed under an Olympus BX 41^®^ light microscope, and photographed using an Olympus DP12^®^ digital camera.

## Results

### Morphology of the Azara’s agouti testis excurrent ducts

**Figure 1:**
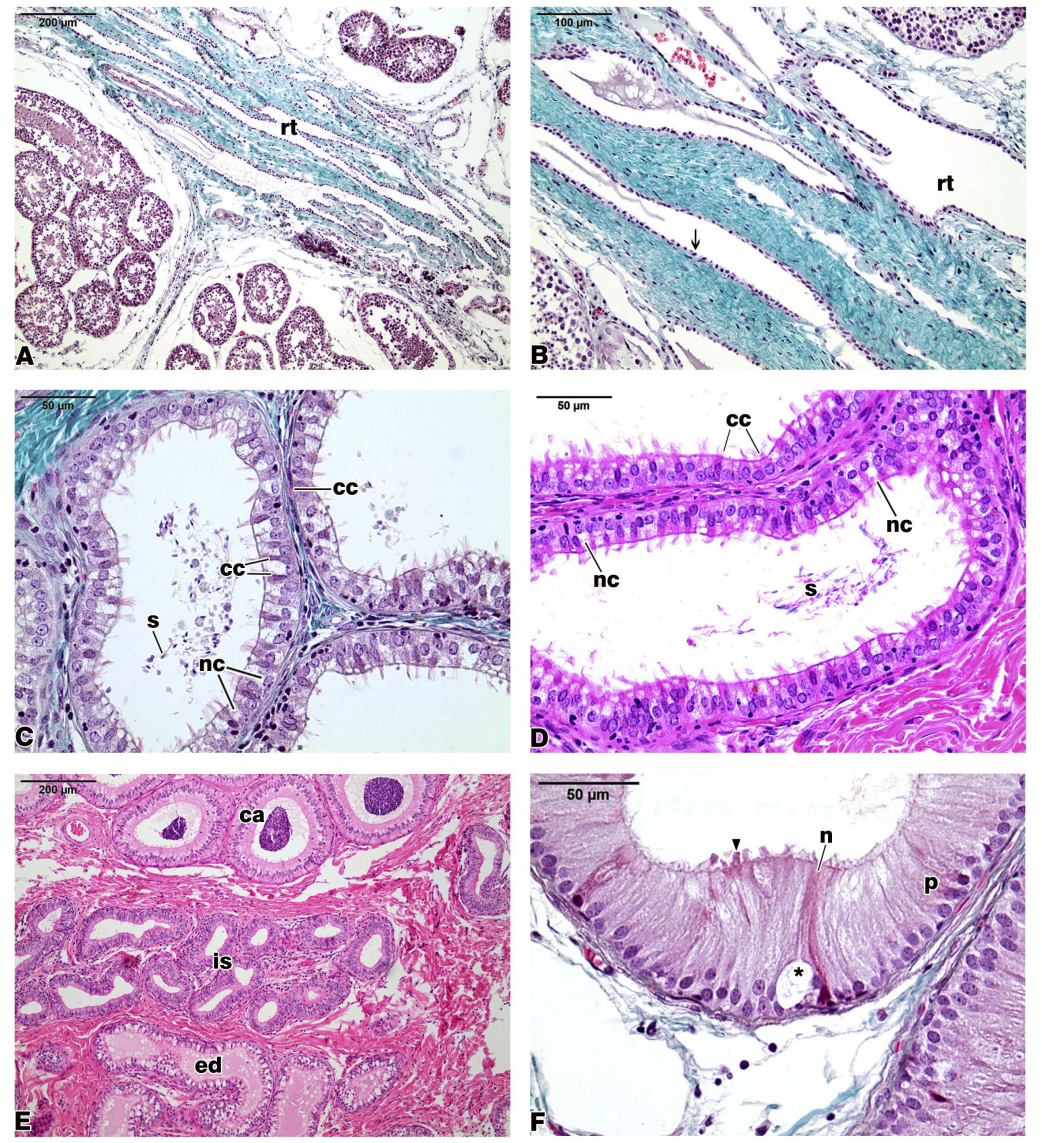
Agouti testis excurrent ducts. Epithelial lining (arrow) of the rete testis (rt) (A,B). Ciliated cells (cc), non-ciliated cells (nc), and spermatozoa (s) in the efferent ducts (C,D). The transition between efferent ducts (ed), initial segment (is), and caput epididymis (ca) (E). The initial segment of the agouti epididymis showing principal (p) and narrows (n) cells, vacuoles (asterisks), and projections of the apical cytoplasm (arrowhead). Masson’s Trichrome (A,B,C,F). Hematoxylin and eosin stain (D,E).

The rete testis (RT) in the Azara’s agouti (Dasyprocta *azarae*) was formed by labyrinthine spaces with axial and labyrinthine characteristics. The RT accompanied the connective axis of the testicular mediastinum, forming a reticular labyrinth of tubules, channels, and interconnected chambers. The interconnected wide channels were lined with a simple cuboidal to the columnar epithellium (Figures 1A and B).

The Azara’s agouti efferent ducts (ED) were a series of tubules connecting the RT to the epididymis. The ED was lined by a simple columnar epithelial lining and was surrounded by connective tissue. The ED cell population consisted of two cell types: ciliated and non-ciliated cells. Intense vacuolation was easily observed in the cytoplasm of the non-ciliated cells in the agouti ED. This vacuolation was not found in the cytoplasm of the ciliated cells. Intraepithelial lymphocytes were occasionally observed in the epithelial lining of the ED. The tubular lumen presented cellular exfoliations and some spermatozoa (Figures 1C, 1D, and 1E).

**Figure 2:**
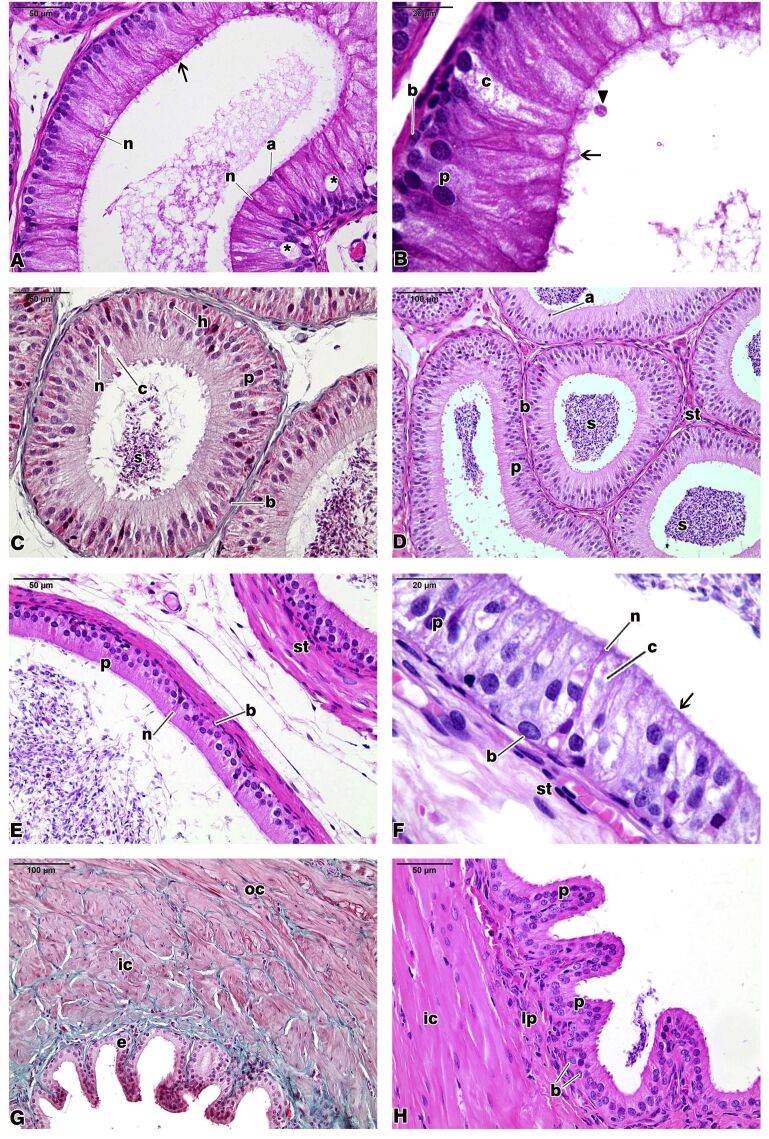
Initial segment (A) of the epididymis, caput epididymis (B,C), corpus epididymis (D), cauda epididymis (E,F), and vas deferens (G,H) in the agouti. Principal (p), basal (b), apical (a), narrow (n), clear (c), and halo (h) cells appear in the epithelium lining, which lies on periductal stroma (st) in the agouti epididymis. The epithelium lining (e) of the agouti vas deferens was composed of principal (p) and basal (b) cells and lies on lamina propria (lp). The muscular coat consisted of an inner longitudinal coat (ic) and another circular one (oc). Note the microvilli (arrows), spermatozoa (s), vacuoles (asterisks), and projections of the apical cytoplasm (arrowhead). Hematoxylin and eosin stain (A,B,C,D,E,F,H). Masson’s Trichrome (G).

The epididymis is a highly coiled duct that connects the ED to the ductus deferens, becoming part of the testis excurrent ducts. A pseudostratified epithelium lined the Azara’s agouti epididymis. This epithelium rested on a delicate basement membrane that was structurally integrated by myoid cells. The tissue surrounding the epididymal duct, denominated peritubular stroma consisted of an arrangement of collagen fibers with fibroblasts, fibrocytes, and smooth muscle cells placed outside the basement membrane of the duct (Figures 1E, 1F, and 2).

The Azara’s agouti epididymis was divided into four histologically defined and sequential segments: initial segment (IS), caput epididymis, corpus epididymis, and cauda epididymis (Figures 1E, 1F, and 2). The IS had a high epithelial lining, a tubular lumen with a regular contour and usually empty, with no material. The lumen was surrounded in its entire length by long microvilli, which protrude from the luminal borders of the epithelial lining (Figures1F,2A, and 2B). Vesicles and vacuoles of various sizes were observed in the epithelial lining of the IS of the agouti epididymis, as well as projections of the apical cytoplasm (Figures1F, 2A, and 2B).

Generally, the caput epididymis showed an oval in outline epithelium with tubular lumen filled with cellular exfoliations and spermatozoa (Figure 2C). Long microvilli and some cytoplasmic projections appeared on the luminal border of the epithelium. The corpus region in the Azara’s agouti epididymis showed regular sections, with shorter and more uniform microvilli than those of the previous regions, and a tubular lumen filled with spermatozoa and cellular exfoliations (Figure 2D). In the cauda epididymis, it was observable that the tubular lumen increased its diameter and that the height of the epithelial lining decreased in comparison with the other epididymal regions. The microvilli that appear at the luminal border of the epithelium were small and uniform, characterizing a “brush border”. The tubular lumen was filled with sperm and cellular exfoliations (Figures 2E and 2F). Visually, it can be observed that the epithelial lining of the Azara’s agouti epididymis decreased its height from the IS towards the cauda epididymis region and that the tubular lumen, in turn, increased its diameter.

The cell population of epididymal epithelium in the Azara’s agouti epididymis comprised principal (P), basal (B), apical (A), clear (C), narrow (N), and halo (H) cells (Figures 1 and 2). In this study, P cells predominated in number and appeared in all the epididymal regions. Apical cells were observed in the IS, caput, and corpus epididymis regions, whereas B cells were found in all epididymal segments studied. Both N and C cells were identified in the IS, in the caput, and cauda epididymis.

Histological analyses showed that the Azara’s agouti vas deferens consisted of an epithelial lining surrounded by a muscular coat and adventitia with a pleated appearance. Sections of the Azara’s agouti vas deferens were lined by a pseudostratified columnar epithelium, whose cellular population consisted of principal and basal cells. The muscular coat consisted of two layers: an inner longitudinal layer and an outer circular one. The tubular lumen was wide and filled with spermatozoa (Figures 2G and 2H).

### Immunolocalization of AQPs in the Azara’s agouti testis excurrent ducts

The immunolocalization of the AQPs in the Azara’s agouti testis excurrent ducts is summarized in Table 1.

**Table 1:** Immunolocalization and intensity of immunoreactions for AQPs -1 and -9 in the agouti testis excurrent ducts.

**Regions**	**AQP1**	**AQP9**
*RT* epithelium	-	-
*ED* apical border	+++	-
*ED* ciliated cells	-	++
*ED* non-ciliated cells	+++	++
*IS* apical border	-	-
*IS* epithelium	-	+++
*CA* apical border	-	-
*CA* epithelium	-	+++
*CO* apical border	-	-
*CO* epitelium	-	+++
*CD* apical border	-	-
*CD* epithelium	-	+++
*VD* apical border	-	-
*VD* epithelium	-	+++

*RT* rete testis, *ED* efferent ducts, *IS* initial segment, *CA* caput epididymis, *CO* corpus epididymis, *CD* cauda epididymis, V*D* vas deferens, *+++* strong reaction, ++ moderate reaction, + weak reaction, - no reaction.

**Figure 3:**
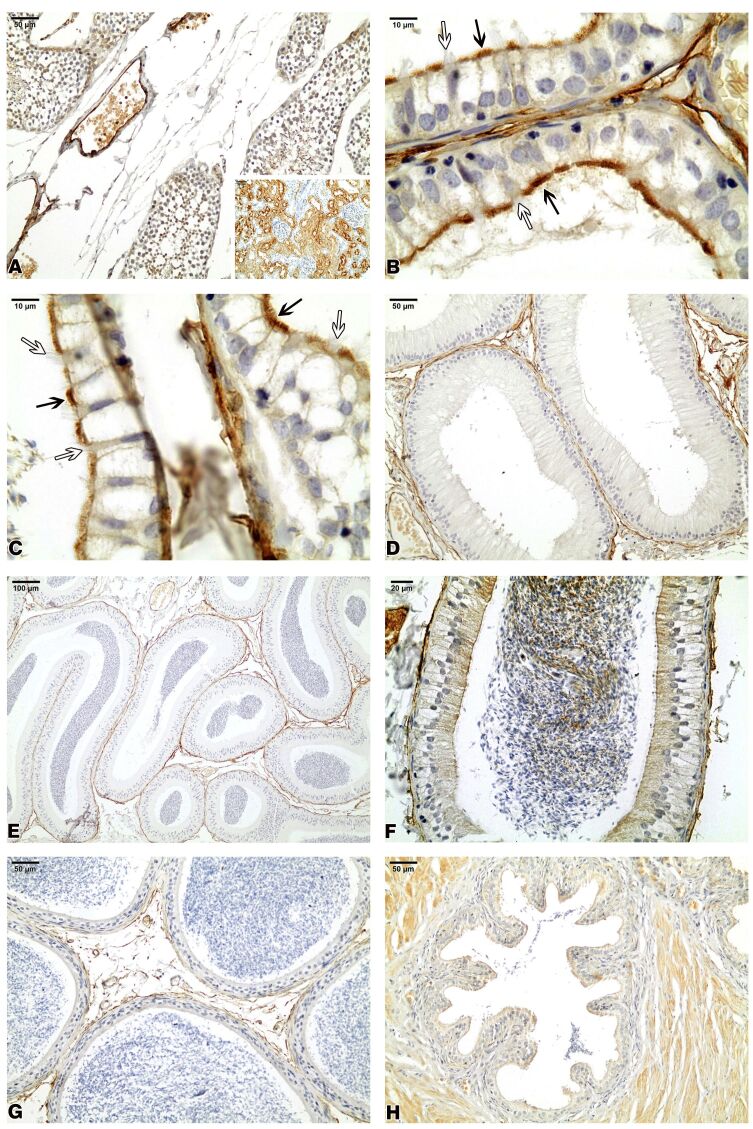
Aquaporin 1 (AQP1) immunohistochemistry in the agouti testis excurrent ducts. No reactivity in the rete testis epithelium (A). AQP1 immunoreactivity is evident in the luminal border of non-ciliated cells (black arrows) and is not detected in the ciliated cells (white arrows) of the efferent ducts (B,C). AQP1 was not immunolocalized in the epithelial cells of the initial segment of the epididymis (D), caput epididymis (E), corpus epididymis (F), cauda epididymis (G), and ductus deferens (H). Inset: adult rat kidney utilized as a positive control for AQP1 immunoreaction.

The RT epithelium lining expressed no reactivity to aquaporin 1 (AQP1) (Figure 3A). Strong AQP1 immunoreactivity was detected in the luminal border of non-ciliated cells of agouti ED. However, AQP1 was not detected in the ciliated cells of the ED (Figures 3B and 3C). No positive reactivity to AQP1 was observed in any region of the Azara’s agouti epididymis since AQP1 was not immunolocalized in the epithelial cells of these epididymal regions. There was AQP1 positive expression only in the endothelial cells of vascular channels located in the peritubular stroma and the interstitium of the epididymal duct. The AQP1 was not also immunolocalized in the epithelial cells of the agouti vas deferens (Figures 3D, 3E, 3F, 3G, and 3H).

**Figure 4:**
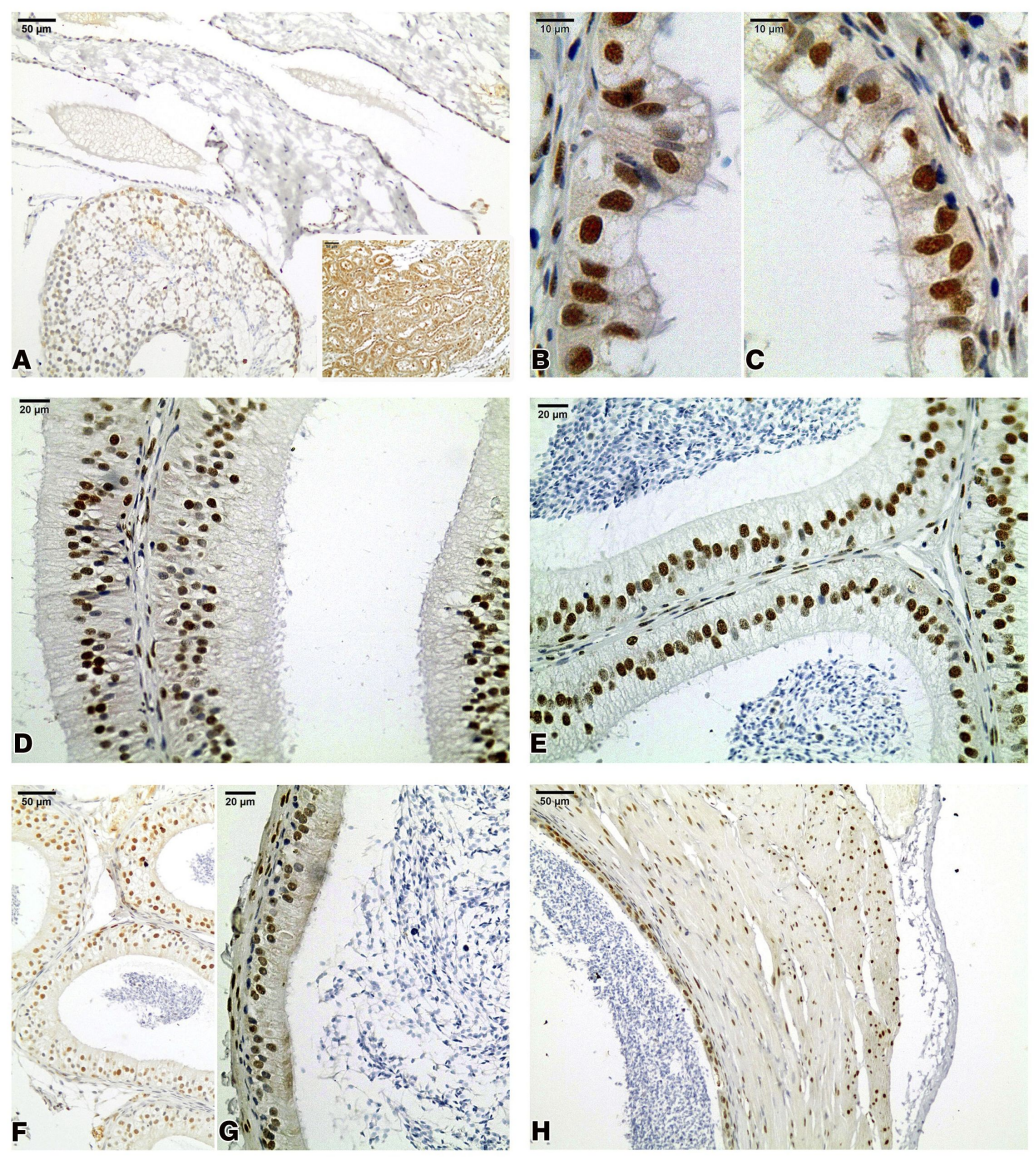
Aquaporin 9 (AQP9) immunohistochemistry in the agouti testis excurrent ducts. No reactivity in the rete testis epithelium (A). AQP9 immunoreactivity was observed in the epithelial cells of efferent ducts (B,C), the initial segment of epididymis (D), caput epididymis (E), corpus epididymis (F), cauda epididymis (G), and vas deferens (H). Inset: adult rat kidney utilized as a positive control for AQP9 immunoreaction.

The RT epithelial lining did not show AQP9-positive reactivity (Figure 4A). A moderate AQP9 immunoreactivity was observed in the epithelial cells of the ED (Figures 4B, 4C). In general, all cell types found in the epididymal epithelium exhibited the same pattern of reactivity in the IS, caput epididymis, corpus epididymis, and cauda epididymis, where a weak to moderate AQP9-positive reactivity was observed in the P, B, and A cells. AQP9 was not detected in the microvilli that appeared in the luminal border of the epithelium of the epididymal regions (Figures 4D, 4E, 4F, and 4G). A similar expression concerning to AQP9 was observed in the epithelium of the vas deferens, with a weak to moderate AQP9-positive reactivity observed in the P and B cells (Figure 4H).

## Discussion

The present study investigated the morphology by light microscopy and immunoexpression of aquaporins (AQPs) 1 and 9 in the rete testis, efferent ducts, epididymis, and vas deferens of the Azara’s agouti (*Dasyprocta azarae*) aiming to fill a gap in the literature about the agouti male reproductive tract. According to Oberska and Michalek (2021), the basic information about AQPs expression is necessary to create “distribution maps” of AQPs in the male reproductive organs, and this will constitute the initial information for further reproductive studies.

The Azara’s agouti rete testis (RT) is a labyrinthine RT, appearing in the mediastinum of the testicle. The RT is cavitary and superficial, in other rodents (Roosen-Runge, 1961). In the guinea pig, the RT is characterized as cavitary, labyrinthine and axial with the arrangement of interconnected epithelial channels; therefore, it presents morphological characteristics of an intermediate type of RT (Beu et al., 1997). The Azara’s agouti RT is characterized by channels lined with a simple cuboidal to columnar epithelium. This type of epithelial lining has been reported in the RT of other mammals (Goyal et al., 1992; Viotto et al., 1993).

The spermatozoa leave the RT through the efferent ducts (ED) that connect the RT to the epididymis. The Azara’s agouti ED is lined by a columnar epithelium that consists of ciliated and non-ciliated cells. Ciliated and non-ciliated cells have been reported in several mammals (Arrighi et al., 1994; Hess, 2002; Ford et al., 2014).

Based on structural and functional parameters, the mouse epididymis is divided into four distinct regions: initial segment (IS), caput, corpus, and cauda epididymis (Robaire and Hinton, 2015). In rodents, the most proximal region of the caput epididymis has a characteristic columnar epithelium with long microvilli, which is different from the caput region, and thus it was called the initial segment (Cornwall et al., 2002). The IS seems to be the most active epididymal segment in mammals and plays an important role in sperm maturation (Cornwall et al., 2002; Cooper et al., 2003). In humans, it appears that there is no distinct IS, and that the proximal portion of the epididymis differs in structure and function when compared to other mammals including experimental animals (Sullivan and Mieusset, 2016). As previously described for the mouse epididymis (Robaire and Hinton, 2015), our histological findings showed that Azara’s agouti epididymis is divided into four regions: initial segment, caput, corpus, and cauda epididymis.

The cellular population of the epididymal epithelium in the Azara’s agouti is composed of principal (P), basal (B), apical (A), clear (C), narrow (N), and halo (H) cells. These same cell types have been observed in the epithelial lining of the mammalian epididymis (Serre and Robaire, 1998; Schimming and Vicentini, 2001; Schimming et al., 2015; Cruceño et al., 2017). Only P, A, B, and C cells were found in the epididymis of the Mexican rodent *Peromyscus winkelmanni* (Lorenzana et al., 2007). In an ultrastructural study of the epididymis and vas deferens of *Dasyprocta* spp., five cell types were found at various stages of sexual development: P, B, H, C, and A cells (Arroyo et al., 2014). These authors did not refer to the presence of N cells, while C cells were only observed after puberty and A cells only in older animals, differently from this study, where P, A, B, C, N, and H cells were detected in all adult animals.

In the Azara’s agouti, P and B cells were found in all epididymal regions. Apical cells were observed in the IS, caput, and corpus epididymis. N and C cells were identified in the IS, caput, and cauda epididymis. The H cells are also present throughout the Azara’s agouti epididymal epithelium. There are differences in the regional distribution of the various cell types present in the epididymis of adult mammals (Arrighi, 2014). Perhaps, the A cells were not visualized in the agouti cauda epididymis region due to the low epithelial height found in this epididymal region (Schimming et al., 2013). N cells have been observed in the IS and C cells in the rat caput, corpus, and cauda epididymis (Robaire and Hermo, 1988; Hermo and Robaire, 2002). The presence of N cells in the IS was reported in the rat and mouse (Robaire and Hinton, 2015) and ram (Schimming et al., 2015) epididymis. H cells are migratory blood cells found throughout the epididymal epithelium (Arrighi, 2014). These cells are identified as lymphocytes, monocytes and/or macrophages, and are, under normal conditions, the primary immune cell in the epididymis (Serre and Robaire, 1998; Robaire and Hinton, 2015).

Thus, the several epididymal cellular types establish a unique and necessary luminal environment for the process of sperm maturation and storage (Breton et al., 2016). In addition, the epididymis also plays an important role in the transport, concentration, and protection of sperm. The cauda epididymis is considered a site for sperm storage, where they complete their maturation process and where they are concentrated (Schimming et al., 2015). The cauda epididymis provides a microenvironment with a metabolic rate and temperature lower than the testicular temperature (Jones et al., 2007), indicating that this epididymal segment is specialized in the storage of spermatozoa.

In the present study, the initial (proximal) segment of the vas deferens was analyzed. The observations confirmed the stratigraphic structure of the vas deferens. The vas deferens is lined by a pseudostratified columnar epithelium with principal and basal cells. The general histological structure of the vas deferens is similar to that described for other mammals. The vas deferens performs functions that go beyond the transport of spermatozoa. Based on the ultrastructure presented by the principal cells, which possess a well-developed Golgi apparatus and endoplasmic reticulum, it is suggested that the principal cells of the mammalian vas deferens play a role in the synthesis and secretion of glycoproteins and steroids (Robaire and Hermo, 1988). In addition, these cells present endocytotic activity, internalizing several substances from the lumen (Andonian and Hermo, 1999). The vas deferens epithelium plays an essential role in the maintaining the luminal environment necessary for the maturation of sperm (Da Silva et al., 2006; Sharma et al., 2020).

The functions performed by the epithelial cells of testis excurrent ducts contribute to a favorable microenvironment for the transport, maturation, and storage of spermatozoa. Transport through cell membranes occurs to establish this microenvironment. Thus, membrane proteins such as the so-called aquaporins (AQPs) probably play an essential role in this transport and, consequently, in forming this microenvironment. The AQPs regulate fluids which is essential for spermatogenesis and posterior spermatozoa transport into the epididymal ducts while maintaining proper ionic conditions for their maturation and storage (Carrageta et al., 2020). AQPs 1 and 9 were not expressed in the epithelium of the Azara’s agouti rete testis, despite indications that mammalian RT epithelial cells exert absorption and secretion functions (Setchell et al., 1994). For these authors, there is reabsorption and modification of a significant part of the seminiferous fluid in the canalicular-cavitary complex of the RT. The secretory role of the RT epithelium has also been described based on findings of light and electron microscopy, including the suggestion of protein-like secretion (Viotto et al., 1993). Thus, RT may be considered a natural biological filter, interposing the barrier between the intra-and extra-testicular excurrent ducts (Roosen-Runge and Holstein, 1978). Perhaps, the processes of fluid regulation in the RT are dependent on other aquaporins isoforms or other types of water channels.

The ED is responsible for transporting the sperm from the testes to the epididymis, while its epithelium lining is responsible for the resorption of about 90% or more of the luminal fluid (Ford et al., 2014). In the Azara’s agouti ED, AQP1 was strongly immunodetected at the luminal border of the non-ciliated cells, whereas AQP9-positive expression was observed in the epithelium. Ultrastructural studies have suggested that non-ciliated cells are involved in the process of endocytosis. Their apical cytoplasm contains an endocytotic apparatus with endocytotic vesicles, apical tubules, endosomes, multivesicular bodies, and secondary lysosomes (Hermo et al., 1988; Hess, 2002; Ford et al., 2014). These structures may explain the intense vacuolization observed in the non-ciliated cells of the Azara’s agouti ED. Studies utilizing markers such as gold particles have demonstrated that non-ciliated cells can reabsorb luminal content (Nakai et al., 2001). Therefore, in addition to transporting the spermatozoa, ED would play an important role in sperm maturation by reabsorbing water, ions, and proteins, which increase the spermatozoa concentration reaching the epididymis (Clulow et al., 1994; Ford et al., 2014). The involvement of the ED in the fluid reabsorption process would explain the intense expression of AQP1 at the luminal border of the non-ciliated cells in the Azara’s agouti ED. This type of immunodetection was also observed in the cat ED (Arrighi et al., 2010).

AQP1 immunoreactivity was not observed in the epithelial lining in the epididymal regions or the vas deferens, except in the blood vessels and epididymal peritubular stroma. This pattern for AQP1 has also been observed in the dog (Domeniconi et al., 2008) and the domestic cat (Arrighi et al., 2010). A positive expression for AQP1 in the endothelium of the blood vessels suggests that this protein channel (AQP1) was probably involved in the removal of water from the tubular spaces in order to contribute to the fluid balance in these tissues (Badran and Hermo, 2002; Arrighi et al., 2010). The expression observed in the peritubular stroma of the epididymis in the Azara’s agouti may suggest that the myoid cells that appear are also involved in removing water from the lumen and transport to the interstitium (Oliveira et al., 2005).

AQP9 was immunodetected in the epithelial cells of the four epididymal regions in the Azara’s agouti epididymis. AQP9 expressions were found in the proximal regions of the epididymis in rams (Schimming et al., 2015) and pigs (Schimming et al., 2017), while there was no expression in the caput of buffalo epididymis (Arrighi et al., 2016). The AQP was detected in the different regions of dog epididymis and vas deferens (Domeniconi et al., 2007). There are suggestions that AQPs 1 and 9 play a pivotal role in the efferent and epididymal ducts concerning the dynamics of secretion and resorption of luminal fluid during transport and sperm maturation (Yeste et al., 2017).

AQP9 immunoreactivity observed in the principal (P) cells can be associated with the activity of secretion and reabsorption of water in the epididymis by this cellular type (Castro et al., 2017; Schimming et al., 2017). The P cells are related to the process of reabsorption of the luminal fluid, endocytosis activities, and secretion of substances such as proteins and glycoproteins (Schimming and Vicentini, 2001; Breton et al., 2016). Both P and C cells are responsible for endocytosis from epididymal luminal fluid (Arrighi, 2014). B cells are considered stabilizing elements of the epithelial lining. However, in recent years, ultrastructural studies have shown that B cells have thin processes that extend along the basement membrane and are directed towards the lumen, becoming indistinguishable from adjacent cells. Based on this morphology and the presence of a solitary cilium projecting into the intercellular spaces, B cells should probably monitor the luminal microenvironment and consequent cell-cell cross-talk, possibly regulating many P cell functions (Arrighi, 2014).

A, C and N cells present intense expression of ATPase in the vacuolar proton pump (V-ATPase) at its luminal border. This suggests that these cell types are involved in the secretion of protons into the lumen, acidifying the intraluminal environment (Breton et al., 1996; Pietrement et al., 2006; Da Silva et al., 2007; Belleannée et al., 2010; Arrighi, 2014; Mandon and Cyr, 2015). This acidification is important for sperm inactivity when stored in the cauda epididymis (Arrighi, 2014). Thus, C cells would maintain a suitable medium for the storage of spermatozoa in a quiescent state during transit through the epididymis (Shum et al., 2009; Menezes et al., 2018). An elaborate network of communication between these epididymal cell types contributes to the regulation of several transport mechanisms in the epididymis of mammals, resulting in the process of sperm maturation (Cheung et al., 2005; Shum et al., 2011; Breton et al., 2016).

The Azara’s agouti vas deferens showed AQP9 immunoreactivity in the epithelial cells of the proximal segment. In the dog, AQP9 staining was also found in the P cells in the proximal region of the duct (Domeniconi et al., 2007). The AQP9 is present in the rat vas deferens, and it is detected throughout the entire length of the duct (Da Silva et al., 2006). Several channels that regulate the fluid composition in the lumen of the rat vas deferens such as AQP9 were studied (Sharma et al., 2020). To these authors, AQP9 immunofluorescence was localized on the luminal surface and along the stereocilia, suggesting that this channel regulates fluid and electrolyte balance in the lumen of the vas deferens.

## Conclusions

Although the distribution of cell types in the epididymal epithelium differs slightly from that described for other species, in general, the morphology presented by the Azara’s agouti testicular excurrent ducts does not differ significantly from that reported for other rodents such as the rat, guinea pig, and *Cuniculus paca*. The results of the immunolocalization of the AQPs confirm that the expression of AQPs is species-specific, due to differences in localization and expression when compared to studies in other mammals and probably, that AQP9 plays a pivotal role in the regulation of fluid and sperm volume regulation in the Azara’s agouti testis excurrent ducts, contributing to spermatozoa concentration and maturation. The knowledge about the expression of AQPs in Azara’s agouti testis excurrent ducts is essential to support future reproductive studies on this animal, since previous studies show that AQPs may be biomarkers of male fertility and infertility.
